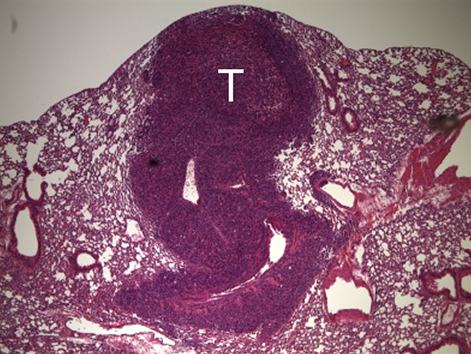# A new mouse model to elucidate mechanisms of metastatic breast cancer

**Published:** 2015-03

Most forms of cancer, including breast cancer, evolve towards metastatic disease, which is very often incurable. Thus, research efforts are directed to advancing knowledge of metastasis mechanisms and to developing new anti-metastasis treatments. However, for breast cancer (which is highly variable in nature), valuable models able to reflect cancer heterogeneity are lacking. In this study, Cameron Johnstone, Yvonne Smith and colleagues used a mouse breast cancer line, designated EO771, derived from a spontaneous mammary tumour of a C57BL/6 mouse. From this line, they subsequently derived a variant that is spontaneously metastatic to the lung, called EO771.LMB. *In vitro* and *in vivo* analyses of EO771 and EO771.LMB revealed that these lines have characteristics of basal-like human breast tumours, a subtype with poor prognosis in humans. In addition, the authors identified important genes – including those that encode matrix metalloproteinase-3 (MMP-3) and parathyroid hormone-like hormone (Pthlh) – whose dysregulation might be causally involved in metastatic dissemination of this type of cancer. This model represents a valuable system to test new therapies for metastatic basal-like breast cancer, with the added advantage of being syngeneic to the C57BL/6 strain in which many transgenic mice are developed.

**Page 237**

**Figure:**